# Caring for Workers’ Health: Do German Employers Follow a Comprehensive Approach Similar to the Total Worker Health Concept? Results of a Survey in an Economically Powerful Region in Germany

**DOI:** 10.3390/ijerph16050726

**Published:** 2019-02-28

**Authors:** Aileen Hoge, Anna T. Ehmann, Monika A. Rieger, Achim Siegel

**Affiliations:** Institute of Occupational Medicine, Social Medicine and Health Services Research, University Hospital Tübingen, Wilhelmstraße 27, 72074 Tübingen, Germany; Aileen.Hoge@student.uni-tuebingen.de (A.H.); Anna.Ehmann@med.uni-tuebingen.de (A.T.E.); Monika.Rieger@med.uni-tuebingen.de (M.A.R.)

**Keywords:** workplace health management, total worker health, workplace health promotion, occupational health and safety, company reintegration management, return to work, cross-sectional survey, Germany

## Abstract

Similar to ‘Total Worker Health’ in the United States (USA), ‘Workplace Health Management’ in Germany is a holistic strategy to protect, promote, and manage employees’ health at the workplace. It consists of four subcategories. While the subcategories ‘occupational health and safety’ and ‘reintegration management’ contain measures prescribed by law, ‘workplace health promotion’ and ‘personnel development’ can be designed more individually by the companies. The present study focused on the current implementation of voluntary and legally required measures of the four subcategories, as well as companies’ satisfaction with the implementation. A total of *N* = 222/906 companies (small, medium, and big enterprises of one German county) answered a standardized questionnaire addressing the implementation of health-related measures, satisfaction with the implementation, and several company characteristics. In the subcategory ‘occupational health and safety’, 23.9% of the companies fulfilled all of the legally required measures, whereas in the category ‘reintegration management’, that rate amounted to 50.9%. There was a positive correlation between company size and the implementation grade, and as well between company size and the fulfilling of measures required by law. Companies tended to be more satisfied with higher implementation grades. Nevertheless, a surprisingly high proportion of the companies with poor implementation indicated satisfaction with the measures’ implementation.

## 1. Introduction

Similar to many other high-income countries, Germany currently faces two trends that have a serious impact on its economy and workforce. First of all, the composition of the working population is shifting toward older age groups, which is a process that will probably be accompanied by an increase in the burden of non-communicable diseases among the workforce [1,2]. Secondly, many branches of the German economy are confronted with an acute shortage of skilled workers and qualified staff, which is a situation that has persisted for years, and recently deteriorated [3,4]. Against this background, stakeholders are increasingly recognizing activities that strengthen the workability and employability of the workforce and promote the good health of workers [2,5]. Thus, it comes as no surprise that during the last few decades, a strategy called ‘workplace health management’ (in German ‘Betriebliches Gesundheitsmanagement’) has gained popularity in Germany [6,7,8]. ‘Workplace health management’ is very similar to the ‘Total Worker Health’ approach in the USA [9,10,11,12,13,14,15,16,17,18,19]. The National Institute for Occupational Safety and Health (NIOSH) defined ‘total worker health’ as activities integrating protection from work-related safety and health hazards with the promotion of injury and illness prevention efforts in order to advance worker well-being [12,13]. The German ‘workplace health management’ approach pursues a similarly holistic strategy. It is commonly defined as the integration and management of all operational processes (in an enterprise) so as to create healthy working conditions and promote the health of its employees [5,20]. Workplace health management can be differentiated into four components or subcategories: (1) occupational health and safety measures, (2) management of the return to work process of employees who have been on long-term sickness absence (in short: ‘reintegration management’), (3) workplace health promotion, and (4) a corresponding personnel development. In Germany, these components differ as to their legal status: whereas many occupational health and safety measures as well as some reintegration management activities are required by law, measures in the areas of workplace health promotion and personnel development are voluntary (cf., in greater detail below).

While the importance of comprehensive workplace health management in Germany seems to be commonly recognized in public discourse, a quite different question is whether and to what extent enterprises actually follow the concept in practice. From several surveys we know that in small and medium enterprises (with up to 250 employees, or—according to another common categorization—with up to 500 employees), workplace health management is often neglected. The ability or willingness to implement workplace health management measures seems to depend linearly on company size. The smaller the company, the less likely it is that a comprehensive workplace health management will be implemented [21,22,23,24]. Small enterprises with up to 50 employees seem to have implementation deficits even with regard to occupational health and safety measures that are required by law [25]. Thus, as to small and medium enterprises (SMEs), the situation in Germany seems to be comparable to the United States (USA) and other European countries [26,27].

In light of these former surveys we wanted to find out the current situation in a German region in which the social and economic environment for health-related measures is comparably good, i.e., clearly above average. If the results of such a survey show that the implementation of workplace health management measures is still as poor as previous surveys suggest, we may conclude and confirm that serious implementation problems persist also within an above-average social and economic environment. Thus, we designed a short survey of health-related measures in small, medium, and big enterprises in the county of Reutlingen (Landkreis Reutlingen). As far as socio-economic strength and population health is concerned, the County of Reutlingen is well above the German average. In 2015, e.g., the unemployment rate in the county was 3.7% (Germany: 6.4%), the average monthly household income per inhabitant amounted to 1946 €, i.e., about 2208 USD (Germany: 1787 €, i.e., about 2028 USD), and the gross domestic product per inhabitant was 38,400 €, i.e., about 43,574 USD (Germany: 36,900 €, i.e., about 41,872 USD) [28]. At the same time, the average life expectancy in the county was 82.69 years (Germany: 80.89 years). At the end of 2015, the county had 282,000 inhabitants. Furthermore, five out of 26 municipalities in the county have been certificated as ‘healthy communities’ because of their commitment to promote physical activity and population health.

In our survey, we addressed only companies that had a minimum size of 10 employees in craft enterprises or 20 employees respectively in non-craft enterprises (cf., further details in the next section). Craft enterprises are enterprises that do not produce industrial mass goods, but generally work to order or provide services on demand (such as carpenters, painters, etc.). As we know from previous studies that the implementation of health-related measures in micro enterprises is very poor or virtually non-existent [21,22,23,24], we concentrated—for economic reasons—on enterprises that had a certain minimum size. Thus, our focus on small, medium, and big enterprises (leaving aside micro, i.e., very small enterprises) and on the county of Reutlingen sets the framework for the following argument. If the degree of implementation of workplace health management measures in the companies we surveyed is good or acceptable, we should not conclude that this is the same (or similar) on average in Germany. On the other hand, if the degree of implementation is poor even in the companies we surveyed, we can conclude that this probably also applies to the German average.

In this context, we will answer the following research questions (RQs):

RQ 1: What is the current state as to the implementation of various workplace health management measures in the companies we addressed in our survey?
RQ 2: Do enterprises generally comply with legal requirements in the areas of occupational health and safety and reintegration management?
RQ 3: What influence does the size of the company have on implementation status?
RQ 4: How satisfied are company representatives with the implementation status as to the above-mentioned four components of workplace health management? How aware are the company representatives of inadequate implementation?

## 2. Materials and Methods

### 2.1. Data Collection

In July 2017, *N* = 906 enterprises in the county of Reutlingen in southwestern Germany were determined as potential respondents of the survey. This number contained all of the enterprises in the county, except for the very small ones: we excluded craft businesses with less than 10 employees and non-craft enterprises with less than 20 employees.

At the end of July and the beginning of August 2017, we sent a standardized questionnaire to these 906 enterprises. Craft businesses received our letter via the local chamber of crafts, which supported the survey; non-craft enterprises received the questionnaire directly from our institute, as we were able to use the complete address data record of the county’s enterprises that was available from a marketing agency (Creditreform [29]). An enclosed leaflet included the request to hand out the questionnaire either to the managing director or to a member of the personnel department. Fourteen days after the first invitation to participate in the survey, a reminder was sent to all of the potential participants, regardless of whether some of them had already returned the questionnaire.

A formal ethical approval from the ethical committee at the University Hospital Tübingen was not required. Study participants were informed that the study was voluntary, and that all of the data were analyzed anonymously.

### 2.2. Questionnaire

The questionnaire was based on previous studies and current literature [24,30,31,32,33]. It was developed, discussed, and formulated in a multidisciplinary team consisting of a specialist in occupational medicine (MAR), a sociologist and public health researcher (AS), and a medical student (AH). After a pretest with *N* = 24 participants (senior employees of the personnel departments of different enterprises of the metal and electrical industry in southwestern Germany), the questionnaire was partially modified and supplemented to ensure good comprehensibility.

Based on a self-developed questionnaire for a similar survey of companies in Constance County that was conducted in 2015 [34], questions covered the implementation status of four categories of health-related measures within the enterprise, referring to the above-mentioned four components of workplace health management. Each category was assessed by several items depicting typical measures (cf., Table 1). Answers regarding the implementation of individual measures in the company within the last two years could be given on a three-point Likert scale (zero = ‘no’, one = ‘no, but in concrete planning’, two = ‘yes’). Hereby, the order of the four categories was as following: workplace health promotion (six items and one possibility for free-text indication), occupational health and safety (seven items and one free-text indication), personnel development (five items and one free-text indication), and reintegration management for employees on long-term sickness absence (eight items and one free-text indication) (cf., Table 1 for all items).

Next, the participants were asked about their satisfaction with the current implementation of the four categories of measures. Here, a four-point Likert scale was used (zero = ‘very dissatisfied’, one = ‘rather dissatisfied’, two = ‘rather satisfied’, and three = ‘very satisfied’).

At the end of the questionnaire, sociodemographic data of the respondents and company characteristics were gathered (branch, number of employees, availability of occupational health and safety experts, and number of employees addressed in reintegration management during the last two years).

### 2.3. Statistical Analysis

For each category of measures a score, ranging from ‘zero’ to ‘10’, was calculated to represent a standardized implementation grade. This score was only calculated if at most one entry per category was missing. A score of ‘zero’ points corresponded to no offered measures and no measures in concrete planning, while a score of ‘10’ stood for the complete implementation of all the listed measures in a given category. The legal requirements in a given category were considered ‘fulfilled’ if all of the legally required measures of that category had been implemented. All seven measures listed in the category ‘occupational safety and health’ were legally required due to regulations in the “Arbeitsschutzgesetz” ([Act on the Implementation of Measures of Occupational Safety and Health to Encourage Improvements in the Safety and Health Protection of Workers at Work]—ArbSchG (1996) [35]), in the “Verordnung über die arbeitsmedizinische Vorsorge” ([Ordinance on Occupational Health Care]—ArbMedVV (2008) [36]), in the “Arbeitssicherheitsgesetz” ([Act on Occupational Physicians, Safety Engineers, and Other Occupational Safety Specialists]—ASiG (1973) [37]), in the “DGUV Vorschrift 1” ([DGUV Regulation 1 “Principles of prevention”] (2013) [38]) and the “DGUV Vorschrift 2” ([DGUV Regulation 2 “Occupational physicians and OSH professionals”] (2011) [39]), and the first two measures listed in the category ‘reintegration management’ were legally required due to the respective regulations in “Book Nine of the Social Code” (Sozialgesetzbuch) (§ 167 SGB “Prevention” [40]) (cf., Table 1).

Rank correlation (Spearman’s r) coefficients were calculated to analyze relationships between ordinal variables (such as, e.g., satisfaction with a given implementation status) and metrically scaled variables or when metrical variables were not normally distributed. Thus, e.g., Spearman’s r was calculated to compare companies of different sizes, which were measured by their number of employees, in terms of adherence of legal requirements (categorized as either ‘yes’/‘fulfilled’ or ‘no’/‘not fulfilled’). To analyze relationships between metrically scaled and normally distributed variables, we calculated Pearson’s correlation coefficients. Coefficients up to 0.3 were classified as low, those between 0.3–0.5 were classified as moderate, and those from 0.5 on were classified as high [41]. The level of significance was set to *p* < 0.05.

As part of a non-responder analysis, responding and non-responding companies were compared concerning their company size. For this purpose, we used an ordinal five-point scale of company size that had been delivered by the Reutlingen Chamber of Crafts for craft enterprises, and thus was available for both responders and non-responders. We proceeded similarly with regard to the non-craft enterprises.

All of the analyses were performed with SPSS, version 24 (IBM Analytics, IBM Corporation, Armonk, NY, USA).

## 3. Results

### 3.1. Participants

The response rate to the questionnaire was all in all 24.5% (*N* = 222/906). On average, there were less than 5% missing values in each category of the questionnaire. The response was above average in medium-sized companies (cf., Table 2) with 101 to 500 employees (31.5% and 32.3%), whereas it was clearly below average in small enterprises with up to 50 employees (22.1%) and in big companies with more than 500 employees (23.1%). Then, the correlation between response and company size seems to be of the inverted u-shaped type. About half of the companies (48.2%) indicated the availability of an occupational health physician, with a range from 29.4% (small companies with up to 50 employees) to 85.0% (companies with 201 to 500 employees). The presence of an occupational safety engineer was reported by 76.8% of all the participating companies (cf., in greater detail in Table 2).

In enterprises with up to 50 employees (the maximum number differs between individual branches due to the respective accident prevention regulation of the respective statutory accident insurance), the employer can participate in a specific occupational health and safety training that entitles him to utilize the service of an occupational safety engineer only when necessary (so-called “Unternehmermodell”). This was indicated by 32/127 enterprises (missing *n* = 5) with up to 50 employees.

As to the sectoral affiliation of the participating companies, almost one third (30.2%; *n* = 67) of the participating companies belonged to the manufacturing industry, and 16.7% (*n* = 37) belonged to the construction industry. Another 15.3% (*n* =3 4) and 14.0% (*n* = 31) can be attributed to services and trade, respectively. The remaining 24% of participating companies were distributed among the following sectors: hospitality industry, agriculture and forestry, maintenance and repair, banking and insurance, transport/storage/communication, public administration, mining and quarrying, education, and energy and water supply.

The sociodemographic characteristics of the responding persons in the companies are shown in Table 3. As to the position of the respondents, 52.7% of these were managing directors, 34.7% were from the personnel department, and 11.7% were other employees (cf., Table 3).

### 3.2. Current State of Implementation of Health-Related Measures in the Companies

In this subsection, we consecutively present the results of the first three research questions (RQ 1, RQ 2, and RQ 3, as explicated in the Introduction).

RQ 1: The average implementation grade of health-related measures in companies as assessed by scores was highest in the category ‘occupational health and safety’ (6.75 points on a scale between zeo and a maximum of 10 points), followed by ‘personnel development’ (6.11 points), ‘reintegration management’ (4.06 points), and finally ‘workplace health promotion’ (3.63 points) (cf., in detail Table 4).

RQ 2: All health-related measures that are required by law were fulfilled by 23.9% (*n* = 53) of the companies in the category ‘occupational health and safety’ and by 50.9% (*n* = 113) in the category ‘reintegration management’.

RQ 3: There is a positive correlation between company size and implementation grade in the four categories of health-related measures. This means for all four categories of health-related measures, the bigger the company, the more measures have been implemented. In the category ‘reintegration management’, the correlation is the most pronounced (Pearson’s r = 0.35, *p* < 0.001), followed by ‘workplace health promotion’ (Pearson’s r = 0.26, *p* < 0.001), ‘occupational health and safety’ (Pearson’s r = 0.23, *p* < 0.001), and ‘personnel development’ (Pearson’s r = 0.21, *p* = 0.002).

There is also a positive correlation between company size and the fulfilling of measures required by law (occupational health and safety: Spearman’s r = 0.35, *p* < 0.001; reintegration management: Spearman’s r = 0.38, *p* < 0.001).

In the next subsection, we present the results of the fourth research question (RQ 4, as explicated in the Introduction).

### 3.3. Satisfaction with Implementation Status

In case important—or even legally required—health-related measures are lacking, it is important to know whether and to what extent these companies are aware of this deficiency before planning any interventions.

In the present survey, company representatives generally tended to be more satisfied with the implementation of a given category of health-related measures the higher the implementation score of their company was in that category. Thus, correlation analyses showed that satisfaction—as measured by the four-point Likert scale—was positively associated with the implementation score value in the categories ‘workplace health promotion’ (Spearman’s r = 0.34, *p* < 0.001), ‘occupational health and safety’ (Spearman’s r = 0.16, *p* = 0.022), ‘personnel development’ (Spearman’s r = 0.21, *p* = 0.002), and ‘reintegration management’ (Spearman’s r = 0.25, *p* < 0.001).

To get further hints on the above-mentioned awareness of company representatives, we furthermore checked how satisfied those company representatives were whose enterprises had a comparably low implementation score in a given category. We defined having a ‘low implementation score’ as belonging to the lowest quartile of the respective scores. In the category ‘workplace health promotion’, *n* = 81 companies (37.3%) belonged to the lowest implementation quartile, and in the category ‘occupational health and safety’, *n* = 62 (28.8%) companies belonged to the lowest implementation. In the category ‘personnel development’, *n* = 58 (26.7%) enterprises belonged to the lowest implementation quartile, while in ‘reintegration management’ this was true for *n* = 57 (26.8%) companies. Within each of these groups of enterprises with a comparably poor implementation of corresponding measures, a substantial proportion of company representatives were nevertheless satisfied (either ‘rather satisfied’ or ‘very satisfied’) with the implementation status (cf., in detail Table 5). With regard to the current situation in the domain ‘workplace health promotion’, *n* = 33 (40.7% of those companies that belonged to the lowest implementation score quartile) company representatives were satisfied. As to the domain ‘occupational health and safety’, *n* = 55 (88.7%) company representatives were satisfied in spite of their comparably poor implementation grade. As to ‘personnel development’, *n* = 39 (67.2%) company representatives were satisfied, despite the relatively poor implementation status of their companies, and regarding the category ‘reintegration management’, *n* = 25 (43.9%) of company representatives were satisfied, although they had a poor implementation record in this category. Thus, a substantial proportion—if not the majority—of ‘under-performing’ enterprises (those belonging to the lowest score quartile) seemed to be satisfied despite a comparably poor implementation.

Turning to the association between satisfaction and the fulfillment of legally required measures in a given domain, the results were as follows (cf., Table 6). Among those companies that did not comply with all of the legal occupational health and safety requirements as assessed in this study (*n* = 155), 92.3% (*n* = 143) were satisfied with the current status of their company’s occupational health and safety implementation. This proportion was nearly as high as within the group of company representatives whose companies fulfilled the listed legal requirements (96.2%). Correspondingly, there was no significant correlation between satisfaction (dichotomously grouped into ‘satisfied’ versus ‘dissatisfied’) and the fulfillment of legally required measures in that domain (Chi^2^ test *p* = 0.383; Fisher’s exact test *p* = 0.522). As to the category of ‘reintegration management’ (cf., Table 6), among those companies that did not comply with all of the listed legal requirements (*n* = 101), 53.5% (*n* = 54) were satisfied with the current situation of their company’s reintegration management implementation. As to this domain, there was a significant but low correlation between satisfaction (grouped into ‘satisfied’ versus ‘dissatisfied’) and the fulfillment of the legally prescribed measures (Spearman’s r = 0.22; *p* = 0.002). Nevertheless, in both domains, a majority of respondents representing companies that did not fully comply with legal requirements were satisfied (either ‘very’ or ‘rather satisfied’); as to the occupational health and safety domain, this majority seemed to be overwhelming (92.3%).

## 4. Discussion

The aim of the study was to provide an assessment of the implementation (RQ 1 and RQ 2) and satisfaction with workplace health management activities (RQ 4) in enterprises in the economically very strong county of Reutlingen. In addition, relationships between company size and implementation (RQ 3) as well as between implementation and satisfaction were to be analyzed and discussed.

### 4.1. Study Design, Questionnaire, Response Rate, and Data Quality

We performed an almost complete cross-sectional survey where only enterprises with less than 10 (craft enterprises) or 20 employees (non-craft enterprises) were not included. Yet, due to the cross-sectional design, no causal relationships can be described.

The questionnaire items were developed to retrieve as many typical health-related measures as possible because of the wide range of measures in workplace health management. The respondents’ low utilization of an offered blank text field for further possible “other measures” that had not been presented as listed items suggests that the lists were practically complete.

The response rate of the survey was 24.5%. The response rate is within the range of the common rates for studies of this type [42,43,44]. The non-responder analysis showed that the response rate was the highest in medium-sized companies, whereas it was lower in both small enterprises (with up to 100 employees) and big companies (with more than 500 employees). An average of less than 5% missing answers indicates a high data quality.

The study results show a large deficit regarding the compliance with legal requirements according to the participants’ indications. Less than 25% of the responding enterprises indicated that their company fulfilled all of the listed legally required measures in the category ‘occupational health and safety’; in the category ‘reintegration management’, about half of the surveyed companies (50.9%) indicated the implementation of all the legally required measures. These comparably low compliance rates might be due to several shortcomings. First of all, companies might be not sufficiently informed about their obligations as employers with regard to all aspects of occupational health and safety (legally required since 1973 [37] and 1996 [35], but with major modifications concerning the defined need for occupational health physicians and occupational safety engineers in 2008 and 2011 [36]) and the implementation of reintegration management (legally required since 2001 [40]). Second, the people who indicated the status of the respective measures in the questionnaire might not have been aware of all the activities implemented in the enterprise. One reason for this could be that some of the activities that were surveyed might be implemented more or less in an implicit manner, but not be spoken of explicitly, especially if the occasion (i.e., an accident or work-related health complaint of an employee) is rather rare. Another reason could be that occupational health physicians, occupational safety engineers, and other experts are available and take care of the implementation without the management noticing much of it. Thus, the respective measures might well be implemented, but not known. Fourth, enterprises are not encouraged strongly enough to follow the legal requirements, as there is not enough compliance monitoring by the respective institutions in Germany (government and statutory accident insurances).

Particularly in the category ‘reintegration management’, small enterprises might not see the need for the implementation of methods of reintegration management, because they may not have needed it yet. Possibly in some small enterprises, individual occupational health and safety measures might be taken now and then according to need, but not on a regular basis [45], which would in part explain the low proportion of companies fulfilling all of the listed occupational health and safety measures. Yet, there is no satisfying explanation for only 29.1% to 85.0% of the study participants indicating the availability of an occupational health physician (cf., Table 2), other than the current shortage of occupational health physicians in Germany [46]. The availability of occupational safety engineers in only 63.0% of the small enterprises (10–50 employees) can well be explained by the regulation that the employer himself can participate in an occupational health and safety training offered by the statutory accident insurance with the consequence that usually no occupational safety engineer is necessary (so-called “Unternehmermodell”). The proportion of only 85.4% of enterprises indicating the availability of an occupational safety engineer in companies with 51–100 employees may either be related to the current lack of occupational safety engineers and other occupational health and safety experts in Germany [47] or due to underreporting, which may also explain the figures reported with regard to occupational health physicians.

Taking these aspects together, the lack of implementation, especially in the area of occupational health and safety, may be somewhat overestimated in this survey. However, the findings do point to the need for supportive measures for a better implementation of legally required measures in German enterprises. The same is true for some measures of workplace health promotion in the majority of enterprises, especially with regard to general, rather than work-related, health (median = 0, cf., Table 1).

Due to the positive correlation between company size and the implementation of the components of workplace health management, we may suppose that the real implementation in all of the companies in the county of Reutlingen is even lower than implied in our study, because very small enterprises—where implementation is generally poor [21,22,23,24]—did not participate in this survey. This assumption applies both to legally required measures and voluntary measures. Furthermore, it should be kept in mind that the county of Reutlingen is a German district with an above-average social and economic environment, as has been shown in the Introduction. Then, we have to assume that in other districts with less favorable economic conditions the situation is probably not better or even worse.

Although there is a positive correlation between satisfaction and implementation grade in the four categories, there is still a surprisingly high satisfaction in enterprises with poor implementation (cf., Table 5 and Table 6). This might indicate that many measures, including those required by law, are not considered necessary (or are not perceived as being required by law). This result, as surprising as it is, needs to be taken into account before planning any interventions to improve the implementation of workplace health management measures.

The results of our study seem to show—once again—that the effectiveness of a top–down approach to the implementation of comprehensive health-related measures in enterprises is rather limited, at least in the context of Western, liberal–capitalist social systems. Even (or just?) in Germany, where there is a long tradition of occupational health and safety legislation, this seems evident. Perhaps a different, less top–down approach is more promising in contemporary Western social systems. The demonstration and publication of success stories of companies that have benefited economically from the implementation of comprehensive health management approaches and the dissemination of corresponding best practice models could possibly stimulate more willingness and motivation on the part of companies to adopt such approaches in the mid and long term.

### 4.2. The Significance of the Study in Comparison with Previous Implementation Research in Germany

Compared with previous studies, this study has several special features. Most previous surveys, particularly those in Germany, were focused on workplace health promotion, while this study differentiates between four categories of workplace health management in order to gain detailed data on each category. The correlation between implementation grade and company size is already well evidenced by literature on Germany as well as other high-income countries [21,22,24,25,26,27,48,49,50]. In addition, our study enables the analysis of new relationships such as the correlation between implementation status and satisfaction of company representatives with their implementation status [30].

To get a holistic overview of the current situation of workplace health management, one must move on from the scope of our survey. It is not only important what companies do, but also how they do it and, first of all, to what extent health-related measures actually reach the employees. Subjective perceptions of working conditions and appreciation of employees by managers play an important role in employee health and well-being in companies, as has been shown for the prevention of psychological and psychosomatic disorders in employees in our recent research [44]. Therefore, further research should also integrate this dimension.

## 5. Conclusions

The implementation of health-related measures among companies of one county in southwestern Germany is heterogeneous. There are major shortcomings regarding compliance with legal requirements, as well as specifically in the domain of occupational health and safety measures. Although there is a positive correlation between implementation and satisfaction, surprisingly many companies are satisfied despite a comparably poor implementation of single measures of workplace health management. These conditions—even in a country where occupational health and safety as well as reintegration management for employees are legally required—must be taken into account before planning interventions to improve workers’ health through a comprehensive approach.

## Figures and Tables

**Table 1:** Surveyed measures (items) regarding workplace health promotion, occupational health and safety, personnel development, and reintegration management.

Categories and Items	Median	Mean	Standard Deviation	Min–Max
Workplace Health Promotion				
Measures to promote and maintain work-related health (e.g., stress management, back health, courses or advice on general workplace health issues)	1	0.96	0.94	0–2
Measures to promote and maintain health that go beyond workplace-related health (e.g., addiction prevention, sports and exercise, healthy nutrition)	0	0.76	0.91	0–2
Employee counseling for psychological complaints	0	0.57	0.87	0–2
Introduction of preventive measures of the German pension insurance (e.g., programs such as Betsi, Balance plus)	0	0.20	0.55	0–2
Info material/brochures on work-related health	2	1.08	0.96	0–2
Info material/brochures on health without a particular reference to work	0	0.82	0.97	0–2
Occupational Health and Safety				
*Occupational medical check-ups for early detection and prevention of work-related disorders*	*2*	*1.34*	*0.92*	*0–2*
*Implementation of occupational health and safety rules (e.g., risk assessment of activities or workplaces, regular instruction of employees according to the Occupational Health and Safety Act)*	*2*	*1.89*	*0.40*	*0–2*
*Health-friendly design of working conditions (e.g., adaptation of the working environment, ergonomic improvement of workplaces, improvement of work processes, organization of working time, adherence of working hours)*	*2*	*1.85*	*0.47*	*0–2*
*Causal analysis of accidents at work and on the way to and from work*	*2*	*1.06*	*0.98*	*0–2*
*Derivation of protective measures on the basis of analyzed accidents at work*	*1*	*1.05*	*0.96*	*0–2*
*Analysis of the causes of work-related complaints by employees*	*2*	*1.13*	*0.95*	*0–2*
*Derivation of measures on the basis of work-related complaints by employees*	*2*	*1.18*	*0.93*	*0–2*
Personnel Development				
Management training/supervision/coaching/consulting (e.g., with regard to mobbing, communication, conflict management)	2	1.28	0.89	0–2
Systematic further training of employees	2	1.64	0.71	0–2
Regular staff appraisals (e.g., for personnel development)	2	1.77	0.54	0–2
Support in reconciling private and professional life (e.g., home office, company kindergarten)	2	1.19	0.96	0–2
Use of demographic counseling (e.g., survey on the age structure of employees, planning strategies to keep older employees healthy, etc.)	0	0.28	0.63	0–2
Reintegration Management				
*Observe the duration of sick leave to notice prolonged and repeated incapacity to work*	*2*	*1.58*	*0.79*	*0–2*
*Procedure for addressing employees with long or repeated incapacities to work*	*2*	*1.22*	*0.90*	*0–2*
Procedure for the inclusion of the health insurance fund in the event of long or repeated incapacity to work	0	0.71	0.90	0–2
Structured approach to the planning of occupational reintegration in the event of long or repeated incapacity for work	2	1.21	0.93	0–2
Appointment of a representative for reintegration management in the company	0	0.58	0.84	0–2
Cooperation with the German pension insurance for benefits for participation in working life	0	0.42	0.79	0–2
Cooperation with the Federal Employment Agency for benefits for participation in working life	0	0.58	0.88	0–2
Contact the joint rehabilitation service center	0	0.24	0.59	0–2

Explications regarding Table 1: Fields in italics: in general legally required according to German laws. The question in the questionnaire had read: ‘Which of the listed measures have taken place in your company in the last two years? (Please also take into account offers that took place outside the company but were (co)financed by the company.)’. Answers were given on a 3-point Likert scale: 0 = ‘no’, 1 = ‘no, but in concrete planning’, 2 = ‘yes’.

**Table 2:** Company characteristics of participating companies according to company size.

Company Size	10–50 Employees	51–100 Employees	101–200 Employees	201–500 Employees	>500 Employees
Number of companies addressed	*N* = 570	*N* = 159	*N* = 89	*N* = 62	*N* = 26
Response (%/*n*)	22.3% *n* = 127	25.8% *n* = 41	31.5% *n* = 28	32.3% *n* = 20	23.1% *n* = 6
Occupational health physician available (%/*n*)*	29.1% *n* = 37	63.4% *n* = 26	78.6% *n* = 22	85.0% *n* = 17	83.3% *n* = 5
Occupational safety engineer available (%/*n*)^‡^	63.0% *n* = 80	85.4% *n* = 35	100.0% *n* = 28	100.0% *n* = 20	100.0% *n* = 6

* Occupational medical check-ups according to the relevant legal regulation (ArbMedVV [36]) (e.g., screen work, handling of hazardous substances, or noisy work places) have to be available to all employees in Germany. According to another regulation [39], an occupational health physician has to be available in all enterprises with more than 50 employees (in some branches, this limit is lower), and in the smaller enterprises in case the employer feels the need for occupational health counseling (so-called “alternative, demand-based supervision”). ‡ An occupational safety engineer has to be available in all enterprises with more than 50 employees (in some branches, this limit is lower), and in the smaller enterprises in case the employer feels the need for occupational health counseling (so-called “alternative, demand-based supervision”). In small enterprises (max. 50 employees), the employer can receive special training with regard to occupational health and safety by the statutory accident insurance in order to reduce the need for support by occupational safety engineers [39].

**Table 3:** Sociodemographic characteristics of respondents.

Characteristic		% (*n*)
Position of respondent	Managing director	52.7% (*n* = 117)
	Member of personnel department	34.7% (*n* = 77)
	Other	11.7% (*n* = 26)
	Missing	0.9% (*n* = 2)
Gender of respondent	Male	54.1% (*n* = 120)
	Female	45.0% (*n* = 100)
	Missing	0.9% (*n* = 2)
Age of respondent (in years)	Mean	50.3
	Median	52.0
	Standard deviation	10.6
	Min–Max	25-82

**Table 4:** Average standardized implementation grade (implementation scale mean) in four categories of health-related measures (total sample, *N* = 222).

Category	Workplace Health Promotion (*n* = 217)	Occupational Health and Safety (*n* = 215)	Personnel Development (*n* = 217)	Reintegration Management (*n* = 213)
Mean	3.63	6.75	6.11	4.06
Standard Deviation	2.87	2.81	2.40	2.75

Explications regarding Table 4: Theoretical range of the standardized implementation grade in all four categories: zero to 10. The ‘*n*’ of the individual columns represents the valid number in each case.

**Table 5:** Satisfaction with implementation status in all enterprises vs. enterprises with poor implementation status (enterprises in the lowest implementation score quartile).

Degree of Satisfaction	Workplace Health Promotion (*N* = 217)	Occupational Health and Safety (*N* = 215)	Personnel Development (*N* = 217)	Reintegration Management (*N* = 213)
Enterprises in the lowest implementation score quartile				
	*n* = 81	*n* = 62	*n* = 58	*n* = 57
Dissatisfied: *n* (%)	38 (46.9)	6 (9.7)	18 (31.0)	20 (35.1)
Satisfied: *n* (%)	33 (40.7)	55 (88.7)	39 (67.2)	25 (43.9)
Missing: *n* (%)	10 (12.3)	1 (2.8)	1 (1.7)	12 (21.1)
Enterprises in the upper three implementation score quartiles				
	*n* = 136	*n* = 153	*n* = 159	*n* = 156
Dissatisfied: *n* (%)	25 (18.4)	7 (4.6)	24 (15.1)	30 (19.2)
Satisfied: *n* (%)	110 (80.9)	145 (94.8)	134 (84.3)	119 (76.3)
Missing: *n* (%)	1 (0.7)	1 (0.7)	1 (0.6)	7 (4.5)

Explication of Table 5: For the sake of clarity, the response categories ‘very dissatisfied’ and ’rather dissatisfied’ were combined to form the ‘dissatisfied’ category, while the response categories ‘very satisfied’ and ‘rather satisfied’ were combined to form the ‘satisfied’ category.

**Table 6:** Satisfaction with implementation status in the domains ‘occupational health and safety’ and ‘reintegration management’ according to compliance with legal requirements in a given domain.

Degree of Satisfaction	Occupational Health and Safety (*N* = 208)	Reintegration Management (*N* = 214)
Enterprises that do not fully comply with legal requirements		
	*n* = 155	*n* = 101
Dissatisfied: n (%)	11 (7.1)	32 (31.7)
Satisfied: n (%)	143 (92.3)	54 (53.5)
Missing: n (%)	1 (0.6)	15 (14.9)
Enterprises that fully comply with legal requirements		
	*n* = 53	*n* = 113
Dissatisfied: n (%)	2 (3.8)	19 (16.8)
Satisfied: n (%)	51 (96.2)	90 (79.6)
Missing: n (%)	-	4 (3.5)

Explication of Table 6: For the sake of clarity, the response categories ‘very dissatisfied’ and ’rather dissatisfied’ were combined to form the ‘dissatisfied’ category, while the response categories ‘very satisfied’ and ‘rather satisfied’ were combined to form the ‘satisfied’ category.
